# Can weekly frequency of plyometric training impair strength and power? A short-term comparison in regional-level jump athletes

**DOI:** 10.3389/fphys.2025.1671750

**Published:** 2025-09-29

**Authors:** Ang Li, Hongbo Zhang, Changwei Peng, Yutong Wu, Jia He

**Affiliations:** ^1^ Geely University of China, Chengdu, China; ^2^ Sichuan Normal University, Chengdu, China

**Keywords:** plyometric frequency, fatigue, sports training, long jumping, DOMS, IMTP, standing long jump

## Abstract

**Objective:**

To compare the short-term effects of two versus three weekly PT sessions on strength and jump performance in competitive jumpers, and to examine associations between delayed onset muscle soreness (DOMS) and week-to-week performance changes.

**Methods:**

Thirty-nine male regional-level high and long jump athletes (17–23 years) completed a 4-week non-randomized observational cohort study. In Week 1 all performed two PT sessions; from Week 2, athletes continued with either two sessions per week (n = 19) or increased to three (n = 20). In Week 4, both groups reduced to two weekly sessions as part of a taper, such that Week-4 outcomes reflect shared recovery rather than continued frequency differences. Assessments at baseline and Weeks 1–4 included isometric mid-thigh pull (IMTP), countermovement jump (CMJ), squat jump (SJ), and standing long jump (SLJ). DOMS was recorded weekly. Mixed-design ANOVAs tested group × time effects, and participant-level correlations examined DOMS associations with performance changes.

**Results:**

Significant main effects of time were found for IMTP, SLJ, CMJ, and SJ (all p < 0.001). The 2×/week group showed steady improvements in IMTP and SLJ, while the 3×/week group experienced early decrements during intensified loading, followed by recovery in Week 4 during the taper phase. Participant-level analyses revealed significant negative correlations between DOMS and ΔIMTP (r = −0.38, 95% CI [–0.53, −0.21]) and ΔSLJ (r = −0.63, 95% CI [–0.73, −0.50]); weaker associations were observed for ΔCMJ (r = −0.21, 95% CI [–0.37, −0.03]) but not for ΔSJ.

**Conclusion:**

Training twice weekly promoted more consistent gains, while three weekly sessions induced transient impairments linked to higher DOMS. Week-4 convergence reflected taper-related recovery rather than sustained frequency effects. Monitoring soreness may help coaches optimize load and recovery in jump athletes.

## Introduction

Plyometric training (PT) is highly relevant for enhancing the physical fitness and performance of high and long jumpers due to its ability to increase explosive strength (Ruggiero and Gruber, 2024), speed, and neuromuscular coordination (Bogdanis et al., 2017)—all critical factors in long jump performance (Koutsioras et al., 2009; Allen et al., 2016). By training the lower limbs to quickly transition from eccentric to concentric contractions, PT improves the stretch-shortening cycle efficiency, allowing jumpers to generate greater force during takeoff (Satkunskiene et al., 2021). This training adaptation can enhance the athlete’s ability to generate force rapidly (Harry et al., 2021); however, jump performance is ultimately determined not by instantaneous power alone, but by the impulse applied during take-off—that is, the product of force and the duration of its application. Greater impulse may enable athletes to achieve longer and more effective jumps, even when peak power output is not maximized. Moreover, the improved motor unit recruitment and reflexive muscle activation promoted by PT can enhance the athlete’s ability to produce rapid, high-intensity movements (Hodgson et al., 2005), essential for acceleration on the runway and the explosive leap that follows (Bogdanis et al., 2017). Recent work has also highlighted the role of SSC-based interventions such as mini-trampoline training in regulating stiffness and enhancing elastic energy return, further emphasizing the importance of mechanical efficiency in jump performance (De Maio et al., 2023; Di Rocco et al., 2023).

Although physical training is an important component of programming, its high-intensity eccentric and concentric muscle actions can induce muscular fatigue, particularly in fast-twitch fibers that are heavily recruited during explosive movements (Drinkwater et al., 2009; Macaluso et al., 2012). Research suggests that this fatigue can be caused by microtrauma in muscle fibers, leading to delayed onset muscle soreness, typically peaking 24–48 h post-exercise (Tian and Miao, 2024). In the eccentric phase of plyometric actions, it is primarily the muscle–tendon unit that lengthens under tension, which contributes most to exercise-induced muscle damage and inflammation (Hody et al., 2019), temporarily reducing muscle strength, impulse generation, and neuromuscular coordination in the following days (Arazi et al., 2016). Additionally, metabolic fatigue from the rapid energy demands of plyometrics may impair performance due to the depletion of phosphocreatine and glycogen stores (Huang et al., 2021). Recovery mechanisms, such as increased blood flow and protein synthesis, gradually restore muscle function over several days (Markus et al., 2021). However, repeated plyometric sessions can lead to adaptation, reducing the severity of fatigue and enhancing recovery rates (Ramírez-Campillo et al., 2015b).

Progressive overload in PT, possibly achieved by increasing the frequency or volume of weekly sessions, can temporarily exacerbate fatigue and impair performance due to the heightened demands on both the musculoskeletal and neuromuscular systems (Skurvydas et al., 2010). This overload can also stress the central nervous system, reducing motor unit recruitment and coordination, temporarily decreasing explosive power and performance (Drinkwater et al., 2009). Studies examining progression strategies in PT (Palma-Muñoz et al., 2021) have shown that gradual increases in frequency or volume may elicit favorable adaptations, but most available evidence remains limited to pre–post designs, overlooking short-term fluctuations during the intervention itself. Additionally, the increased frequency allows less time for recovery, resulting in persistent fatigue and decreased energy stores, further impairing performance in subsequent sessions (Ramírez-Campillo et al., 2015b). However, with proper recovery and adaptation over time, the athletes may become more resilient, leading to improved power, endurance, and overall athletic performance (Ramírez-Campillo et al., 2015b).

Although previous studies have compared continuous PT programming with progressive overload PT (Ramírez-Campillo et al., 2015a; Palma-Muñoz et al., 2021), in which volume and/or training frequency are gradually increased, most experimental research has focused primarily on pre- and post-intervention outcomes. Few studies have examined the effects of programming throughout the intervention period itself (Ramírez-Campillo et al., 2015a; Palma-Muñoz et al., 2021). These studies often overlook the immediate effects of progressive training load increments during the intervention. Examining these effects could help the scientific community better understand fatigue and recovery patterns, while providing coaches with insights to detect performance declines and optimize periodization. Such strategies may prevent accumulated fatigue and reduce the risk of impairments that could compromise technical performance or training goals. To address this gap in the literature and considering the scientific and practical relevance of such an approach, the current study adopted a prospective cohort design. It followed two groups of male jumpers: one group continued with two PT sessions per week (2x week), and a second group increased to three sessions per week (3x week). To compare the effects of different weekly training frequency approaches, weekly comparisons between the groups were conducted over 4 weeks. Muscular readiness was assessed through jump tests and maximal strength tests, while the potential impact of increasing training frequency in PT was compared. As a secondary objective, we aimed to analyze how delayed onset muscle soreness (DOMS) may be related to fluctuations in muscular test results over the weeks.

## Methods

### Design and setting

This investigation is a non-randomized observational cohort to examine the effects of training frequency on muscular performance. The research team did not intervene in the assignment process but simply monitored the participants and conducted assessments throughout the study period. Specifically, the study compared two groups: one that maintained a standard PT regimen and another that increased their PT sessions to three per week. The duration of the study spanned 4 weeks, with participants undergoing assessments once each week. Evaluations were conducted at the beginning of each week, 48 h after the final training session, to ensure adequate recovery (Table 1). All assessments were carried out under consistent environmental conditions: indoors at 5:00 p.m., with controlled temperature (ranging from 20.5°C to 21.5°C) and relative humidity maintained between 52% and 55%. In the initial week, all participants followed the same routine of two PT sessions per week, which mirrored the structure they had been adhering to over the previous month. This first week served as the baseline for subsequent comparisons. Beginning in week two, participants were divided into two distinct groups. One group continued with the two-session weekly format, while the other transitioned to a higher frequency, engaging in PT 3×/week. Athlete progress and responses were closely monitored throughout the intervention period.

**TABLE 1:** Weekly plyometric training and testing schedule for the 2×/week and 3×/week groups.

Week	Group	Monday	Wednesday	Thursday	Friday	Following Monday (start of next cycle)
Week 1 (baseline)	Both groups	PT Session 1	–	PT Session 2	–	Testing (48 h after last session)
Week 2	2×/week group	PT Session 1	–	PT Session 2	–	Testing (48 h after last session)
	3×/week group	PT Session 1	PT Session 2	–	PT Session 3	Testing (48 h after last session)
Week 3	2×/week group	PT Session 1	–	PT Session 2	–	Testing (48 h after last session)
	3×/week group	PT Session 1	PT Session 2	–	PT Session 3	Testing (48 h after last session)
Week 4 (taper)	Both groups	PT Session 1	–	PT Session 2	–	Testing (48 h after last session)

#### Participants

Although the present investigation was conducted as a non-randomized observational cohort, the *a priori* power analysis was performed using a two-group (2×/week vs. 3×/week) repeated-measures framework with five test points (baseline, Weeks 1–4). This approach was selected to conservatively estimate the sample size required to detect a group × time interaction on the isometric mid-thigh pull test (IMTP). Power analysis assumed a small–medium interaction effect of Cohen’s f = 0.20 (≈ηp^2^ ≈ 0.038), two-tailed α = 0.05, power (1–β) = 0.80, within-subject correlation r = 0.60, and a conservative nonsphericity correction ε = 0.75. Under these assumptions, the required total N ≈ 38 (≈19 per group), which we rounded to a practical target of N ≈ 39–40 to allow minimal attrition. Calculations were performed with G*Power (version 3.1 or later) using F tests ANOVA: Repeated measures, between–within interaction, with: number of groups = 2, measurements = 5, effect size f = 0.20, α = 0.05, power = 0.80, correlation among repeated measures = 0.60, and nonsphericity correction ε = 0.75.

To be eligible for inclusion in the study, athletes had to meet the following criteria: (i) be a high or long jumper; (ii) have at least 2 years of event-specific training experience; (iii) participate in all scheduled assessment sessions; and (iv) follow the training prescribed by their club coaches throughout the study period. Athletes were excluded if they: (i) were injured during the study period; (ii) were using performance-enhancing substances; or (iii) engaged in any training outside of what was prescribed by their athletics club coaches.

Thirty-nine regional-level high and long jump athletes (age: 19.2 ± 2.3 years; height: 179.2 ± 3.1 cm; weight: 72.9 ± 4.1 kg; BMI: 22.7 ± 1.0) were recruited from a training center. This was a 4-week, non-randomized observational cohort conducted in a real-world setting. Group allocation was determined by the athletes’ personal coaches according to their periodization plans: one group continued plyometric training twice weekly (2×/week, n = 19), while the other increased to three sessions per week (3×/week, n = 20). This design provided a practical opportunity to evaluate the effects of training frequency under real coaching conditions.

Baseline characteristics revealed no significant between-group differences were observed for age (2×/week: 18.9 ± 2.1 vs. 3×/week: 19.4 ± 2.5 years, p = 0.545), stature (179.1 ± 3.1 vs. 179.4 ± 3.2 cm, p = 0.730), body mass (73.2 ± 4.1 vs. 72.6 ± 4.1 kg, p = 0.618), or BMI (22.8 ± 0.9 vs. 22.5 ± 1.0 kg/m^2^, p = 0.359). Likewise, baseline performance outcomes did not differ: IMTP (24.7 ± 2.1 vs. 25.1 ± 2.7 N.kg^-1^, p = 0.648), CMJ (39.3 ± 3.2 vs. 38.7 ± 2.8 cm, p = 0.504), SJ (38.8 ± 3.2 vs. 38.2 ± 2.8 cm, p = 0.501), and SLJ (208.1 ± 16.5 vs. 212.1 ± 18.6 cm, p = 0.483).

Ethical approval for this study was obtained from the Institutional Review Board of Sichuan Normal University (Approval ID: 2025LS0058). Research procedures were fully explained to all participants and, when applicable, to their legal guardians. Written informed consent was obtained prior to participation: individuals aged 18 years and older provided their own consent, while for those under 18 years, consent was obtained from a parent or legal guardian. All procedures were conducted in accordance with the ethical principles of the Declaration of Helsinki for research involving human participants.

#### Plyometric training

On average, athletes trained 5 days per week, combining strength and conditioning, technical skill development, and jump-specific exercises tailored to their event demands. In addition to the prescribed PT, their weekly routines included: (a) strength training (2 sessions, ∼60–75 min, emphasizing Olympic lifts, squats, and accessory lower-limb exercises), (b) sprint and acceleration drills (1–2 sessions, ∼30–45 min), (c) technical jump practice (2 sessions, ∼60 min, focusing on take-off mechanics and approach rhythm), and (d) general conditioning (once weekly, ∼30 min).

PT was integrated into this routine during the second and fourth training days of the week, with the 2×/week group completing two sessions and the 3×/week group adding a third session scheduled 48 h later. Each PT session incorporated both vertical and horizontal movements. The vertical component included single-leg pogo jumps (1 × 10 repetitions per leg, 20 contacts), single-leg butt kicks (1 × 10 per leg, 20 contacts), single-leg tuck jumps (1 × 10 per leg, 20 contacts), and moving single-leg cycles (3 × 5 per leg, 30 contacts), totaling ∼90 vertical contacts. The horizontal component comprised bounds for distance (4 × 5 per leg, 40 contacts), alternate-leg bounds (3 × 10, 30 contacts), and rhythmical horizontal hops (3 × 10, 30 contacts), totaling ∼100 horizontal contacts. Thus, each PT session involved ∼190 ground contacts, performed on track or grass surfaces, following a standardized 20-min dynamic warm-up. Rest intervals were structured as 3 min between exercises/sets and 1 min between unilateral efforts.

Progression was introduced differently for each group. In the 2×/week group, weekly plyometric exposure increased gradually from ∼380 contacts in Week 1 to ∼420–450 in Week 3, before tapering to ∼300 in Week 4. In the 3×/week group, the additional session not only raised total weekly frequency but also accelerated cumulative load, with volumes rising from ∼570 contacts in Week 1 to ∼630–650 in Week 3, then tapering to ∼450 in Week 4. Importantly, per-session structure (exercise types, set–rep schemes, rest intervals, and surfaces) remained equivalent between groups; therefore, the higher total load in the 3×/week group was attributable to training frequency rather than increased per-session intensity. Across both groups compliance exceeded 95%, and no injuries or adverse events were reported.

For comparison, widely cited NSCA-based guidelines (Haff and Triplett, 2015) recommend ∼80–100 (beginner), ∼100–120 (intermediate), and ∼120–140 (advanced) contacts per session; our per-session dose therefore exceeds the “advanced” range, consistent with a high-volume prescription. In athlete studies, effective programs often use ∼80–100 contacts per session (≈160–200 per week at 2×/week) or ∼140–240 per week in soccer samples (Bouguezzi et al., 2020; Moran et al., 2024); by contrast, our weekly totals are higher, though still within the scope of high-volume protocols reported in team-sport literature when progression and recovery are appropriately controlled.

##### Assessments

All participants completed their assessments in the afternoon (4 p.m.) to maintain uniform testing conditions. Prior to each evaluation session, athletes observed a 48-h rest interval. Testing was conducted weekly during weeks 1 through 4 of the study period. The assessment followed a consistent and standardized procedure: participants began with a warm-up that included 5 min of jogging, followed by 10 min of lower limb mobility exercises, and concluded with 10 min of drills focused on muscle power. After warming up, athletes performed the IMTP to measure maximal strength, followed by jump tests in a fixed order (SJ, CMJ, SLJ). A fixed order was maintained across all sessions to ensure standardization between groups. Three-minute rest intervals and familiarization attempts were used to minimize fatigue.

##### Isometric midthigh pull test

During the IMTP test, athletes gripped a fixed bar secured with weightlifting straps to prevent slipping during force exertion. Drawing on their weightlifting experience, participants adopted a stance and hand position similar to the second pull phase of a power clean. Upon the verbal countdown “3, 2, 1, Pull!“, they were instructed to pull the bar with maximum effort and speed while driving their feet firmly into the ground. Hip and knee angles were measured using a handheld goniometer, averaging 143° ± 3° and 146° ± 3°, respectively, to ensure consistent positioning. The grip location on the bar was marked with tape for each athlete to maintain uniformity across attempts. The test setup included a power rack, allowing the bar to be comfortably positioned above a force plate (ForceDecks, Vald Performance, Australia). Force-time data were sampled at 1,000 Hz and processed in the ForceDecks software. Signals were low-pass filtered at 20 Hz using a fourth-order Butterworth filter. Pull onset was defined as the point where vertical force exceeded the baseline value (mean force in the 200 m prior to initiation) by ≥ 5 standard deviations.

Before formal testing, each athlete performed a familiarization trial. This was followed by three maximal effort trials, each separated by 1 min of rest. During these trials, participants sustained maximal force output for 5 seconds. The peak force values normalized by body weight (N/kg) were averaged across the three attempts and recorded for analysis at each assessment point. The average within-athlete coefficient of variation across repeated trials in the same session was 3.8%.

#### Squat jump test

Participants performed the standard bilateral squat jump (SJ) on a force plate (ForceDecks, Vald Performance, Australia). They were instructed to descend to a comfortable squat position, approximately at a 90° knee angle, with their hands resting on their hips. Upon hearing the verbal cue “3, 2, 1, jump!”, the athletes executed a vertical jump, aiming to jump as high and as quickly as possible. Throughout the jump, they were required to keep their hands on their hips, fully extend their knees during flight, and land simultaneously on both feet.

At each testing session, athletes first completed a familiarization jump, followed by three maximal effort attempts, with 1 min of rest between each. The mean values of jump height (cm), from the three jumps were recorded and used for subsequent comparisons over the testing weeks. Across repeated trials within the same session, the mean within-athlete coefficient of variation was 4.3%.

#### Countermovement jump test

The countermovement jump (CMJ) test was also performed on a ForceDecks force plate (Vald Performance, Australia), with jump height determined using the impulse–momentum method. Participants stood upright with hands on hips and, upon the command “3, 2, 1, jump!“, executed a rapid downward squat to roughly 90° knee flexion before immediately jumping as high as possible. Standardized instructions required participants to keep their hands on their hips, avoid any arm swing, fully extend their knees during flight, and land with both feet simultaneously. All jumps were performed in training footwear on the same surface across sessions. Each testing session included one familiarization trial, followed by three maximal effort jumps, separated by 1-min rest intervals. The mean jump height (cm) from the three attempts was calculated and used for subsequent comparisons across weeks. The within-athlete coefficient of variation, averaged across repeated trials conducted in a single session, amounted to 4.5%.

#### Standing long jump

The standing long jump (SLJ) test was conducted with athletes positioned on a marked flat surface. Participants stood with their feet shoulder-width apart behind the starting line, and upon receiving the instruction “3, 2, 1, Jump,” they were directed to jump forward as far as possible, using a two-foot takeoff and landing simultaneously on both feet. Arms were allowed to swing naturally to assist the jump.

Each assessment session started with a familiarization attempt. Afterward, athletes completed three maximal jumps with a 1-min rest between each effort. The longest jump distance (cm) from the three trials was recorded and used for comparison across the different testing sessions.

## Delayed onset muscle soreness

A 7-point Likert-type scale (0–6) adapted from the Hooper questionnaire (Haddad et al., 2013) was used to assess delayed onset muscle soreness (DOMS). In this adapted version, 0 = no discomfort, 1 = very slight soreness, 2 = mild soreness not affecting movement, 3 = moderate soreness noticeable during activity, 4 = considerable soreness that causes some difficulty in training, 5 = severe soreness limiting training quality, and 6 = extreme soreness preventing normal movement or training execution. The rating focused specifically on the lower limbs as a whole (quadriceps, hamstrings, gluteal muscles, and calves), reflecting the localized demands of PT. Athletes were instructed to consider their overall lower-limb soreness rather than isolating individual muscle groups. Assessments were performed once per week, immediately before the scheduled performance testing session, ensuring consistency across time points. Minor modifications were made to the original English descriptors of the Hooper scale to emphasize muscle soreness rather than general wellbeing. Athletes completed the form under supervision of the research team, and the adapted scale was used given its established applicability in monitoring recovery and training load.

## Statistical procedures

The data were tested for normality using the Shapiro–Wilk test, with a significance threshold of p > 0.05. Homogeneity of variances was assessed with Levene’s test, also considering p > 0.05 as indicating equal variances. A mixed-design ANOVA was employed to investigate the effects of time, group, and their interaction. Sphericity was assessed with Mauchly’s test, and when violated, Greenhouse–Geisser corrections were applied. Effect sizes were calculated using partial eta squared (η^2^_p_) for the ANOVA comparing pre- and post-intervention measurements. For analyses reporting ηp^2^, effect sizes were interpreted according to conventional thresholds: small (≥0.01), medium (≥0.06), and large (≥0.14) (Cohen, 1988). Four performance outcomes (IMTP, CMJ, SJ, and SLJ) were analyzed as co-primary variables, given their complementary representation of neuromuscular performance. To control Type I error, Bonferroni corrections were applied for all *post hoc* pairwise comparisons within each outcome.

To explore the relationship between delayed onset muscle soreness (DOMS) and performance adaptations, correlations were performed at the participant level. For each athlete, week-to-week changes (Δ) in IMTP, CMJ, SJ, and SLJ were calculated relative to the prior assessment, and these values were paired with the DOMS scores recorded for the same week. Pearson product–moment correlations were then computed using all available participant-week observations (Weeks 2–4), providing individual-level associations between DOMS and performance fluctuations. Ninety-five percent confidence intervals for correlation coefficients were derived using Fisher’s z-transformation. Statistical analyses were conducted in JASP (version 0.18.3, University of Amsterdam) and in Python (v3.11). Statistical significance was set at p < 0.05.

## Results

Table 2 summarizes the mean and standard deviation for the IMTP, CMJ height, SJ height, and SLJ for the two groups over four consecutive weeks.

**TABLE 2:** Mean and standard deviation (SD) the IMTP, CMJ height, SJ height, and SLJ for the two groups over four consecutive weeks.

Measure	Group	Week 1 mean (SD)	Week 2 mean (SD)	Week 3 mean (SD)	Week 4 mean (SD)
IMTP (N·kg^-1^)	2x/week	24.71 (2.11)	24.94 (2.25)	25.02 (2.17)	25.29 (2.18)
	3x/week	25.08 (2.69)	24.59 (2.75)	23.59 (2.75)	25.23 (2.56)
IMTP absolute (N)	2x/week	1809.6 (184.5)	1826.4 (193.0)	1832.0 (191.8)	1851.3 (185.9)
	3x/week	1824.3 (263.9)	1788.0 (264.0)	1715.4 (261.0)	1835.1 (251.2)
CMJ (cm)	2x/week	39.29 (3.15)	39.32 (3.03)	39.75 (2.84)	36.35 (3.07)
	3x/week	38.66 (2.82)	38.23 (2.94)	37.42 (2.86)	36.36 (2.96)
SJ (cm)	2x/week	38.81 (3.22)	38.82 (3.09)	39.25 (2.91)	35.86 (3.10)
	3x/week	38.16 (2.85)	37.71 (2.99)	37.28 (2.87)	35.85 (2.97)
SLJ (cm)	2x/week	208.13 (16.46)	208.69 (15.78)	210.12 (16.10)	212.06 (16.27)
	3x/week	212.12 (18.62)	209.70 (18.41)	203.59 (18.75)	213.40 (18.57)

Values are mean (SD). CMJ, and SJ, represent the mean of three maximal attempts; IMTP, isometric mid-thigh pull; CMJ, countermovement jump; SJ, squat jump; SLJ, standing long jump.

**FIGURE 1:**
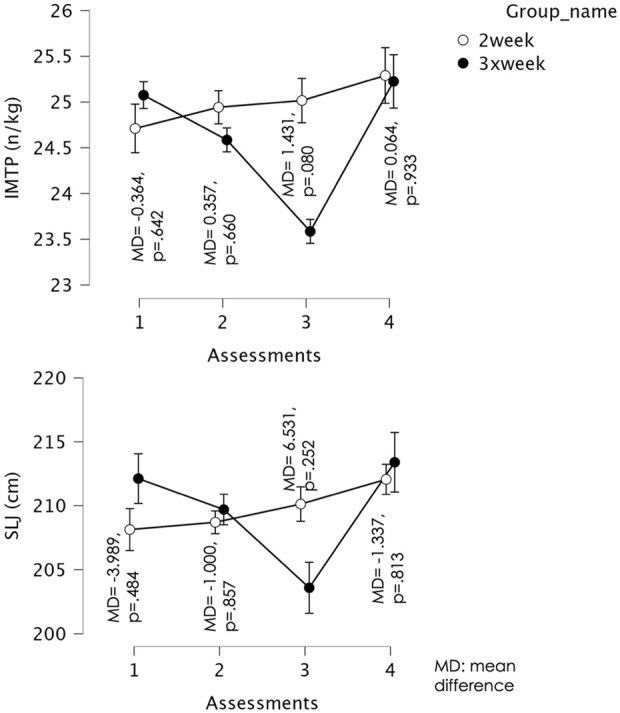
Mean and confidence intervals (CI) at 95% for the isometric mid-thigh pull test (IMTP) and standing long jump (SLJ) values over the course of the assessments. Error bars represent (95% CI). Circles are group means.

For IMTP, Mauchly’s test indicated that the assumption of sphericity was violated (W = 0.364, χ^2^ (5) = 36.12, p < 0.001); therefore, Greenhouse–Geisser corrections were applied (ε = 0.686). The repeated measures ANOVA revealed a significant main effect of time, F (2.06, 76.20) = 28.23, p < 0.001, partial η^2^ = 0.433, and a significant time × group interaction, F (2.06, 76.20) = 26.49, p < 0.001, partial η^2^ = 0.417. For the 2×/week training group, there was no significant difference in IMTP between Week 1 and Week 2 (Mean Difference = −0.232, p = 0.350) or between Week 1 and Week 3 (Mean Difference = −0.305, p = 0.233). However, a significant increase was observed from Week 1 to Week 4 (Mean Difference = −0.579, p = 0.013). No significant differences were found between Week 2 and Week 3 (Mean Difference = −0.074, p = 1.000) or between Week 2 and Week 4 (Mean Difference = −0.347, p = 0.348), but Week 4 values were significantly greater than Week 3 (Mean Difference = −0.274, p = 0.013). For the 3×/week training group, IMTP decreased significantly from Week 1 to Week 2 (Mean Difference = 0.490, p < 0.001) and from Week 1 to Week 3 (Mean Difference = 1.490, p < 0.001). No significant difference was observed between Week 1 and Week 4 (Mean Difference = −0.150, p = 1.000). IMTP also declined from Week 2 to Week 3 (Mean Difference = 1.000, p < 0.001), before increasing significantly between Week 2 and Week 4 (Mean Difference = −0.640, p = 0.004) and between Week 3 and Week 4 (Mean Difference = −1.640, p < 0.001). These results indicate an initial impairment followed by recovery during the taper week. Figure 1 illustrates the comparisons of IMTP between groups across testing weeks.

For SLJ, Mauchly’s test indicated that the assumption of sphericity was violated (W = 0.376, χ^2^ (5) = 34.92, p < 0.001); therefore, Greenhouse–Geisser corrections were applied (ε = 0.701). The repeated measures ANOVA revealed a significant main effect of time, F (2.10, 77.77) = 19.06, p < 0.001, partial η^2^ = 0.340, and a significant time × group interaction, F (2.10, 77.77) = 16.53, p < 0.001, partial η^2^ = 0.309. For the 2×/week training group, there was no significant difference in SLJ between Week 1 and Week 2 (Mean Difference = −0.568, p = 1.000), Week 1 and Week 3 (Mean Difference = −1.995, p = 0.902), or Week 2 and Week 3 (Mean Difference = −1.426, p = 0.883). However, performance improved significantly between Week 1 and Week 4 (Mean Difference = −3.932, p = 0.040) and between Week 2 and Week 4 (Mean Difference = −3.363, p = 0.033), with Week 4 showing greater SLJ values. No significant change was observed between Week 3 and Week 4 (Mean Difference = −1.937, p = 0.510). For the 3×/week training group, SLJ decreased significantly from Week 1 to Week 2 (Mean Difference = 2.420, p = 0.005) and from Week 1 to Week 3 (Mean Difference = 8.525, p < 0.001). No difference was found between Week 1 and Week 4 (Mean Difference = −1.280, p = 1.000). SLJ also declined from Week 2 to Week 3 (Mean Difference = 6.105, p < 0.001), before recovering between Week 2 and Week 4 (Mean Difference = −3.700, p = 0.012) and between Week 3 and Week 4 (Mean Difference = −9.805, p < 0.001), with Week 4 values significantly greater than those of the intensified training weeks. Figure 1 illustrates the comparisons of SLJ between groups across the different assessment moments.

**FIGURE 2:**
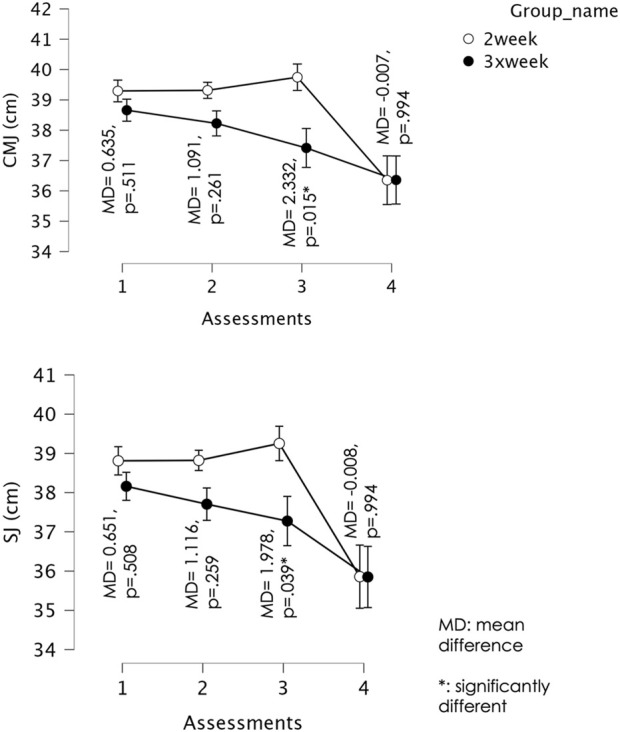
Mean and confidence intervals (CI) at 95% for the countermovement jump test (CMJ) and squat jump (SJ) values over the course of the assessments. Error bars represent (95% CI). Circles are group means.

For CMJ, Mauchly’s test indicated that the assumption of sphericity was violated (W = 0.191, χ^2^ (5) = 59.12, p < 0.001); therefore, Greenhouse–Geisser corrections were applied (ε = 0.531). The repeated measures ANOVA revealed a significant main effect of time, F (1.59, 58.94) = 43.77, p < 0.001, partial η^2^ = 0.542, and a significant time × group interaction, F (1.59, 58.94) = 7.17, p = 0.003, partial η^2^ = 0.162. For the 2×/week training group, there was no significant difference in CMJ height between Week 1 and Week 2 (Mean Difference = −0.021, p = 1.000), Week 1 and Week 3 (Mean Difference = −0.453, p = 0.965), or Week 2 and Week 3 (Mean Difference = −0.432, p = 0.250). However, performance declined significantly between Week 1 and Week 4 (Mean Difference = 2.942, p < 0.001), Week 2 and Week 4 (Mean Difference = 2.963, p < 0.001), and Week 3 and Week 4 (Mean Difference = 3.395, p < 0.001), with Week 4 showing lower CMJ height. For the 3×/week training group, there was no significant change between Week 1 and Week 2 (Mean Difference = 0.435, p = 0.205). However, CMJ height decreased significantly from Week 1 to Week 3 (Mean Difference = 1.245, p = 0.002) and from Week 1 to Week 4 (Mean Difference = 2.300, p < 0.001). Performance also declined between Week 2 and Week 3 (Mean Difference = 0.810, p = 0.001) and between Week 2 and Week 4 (Mean Difference = 1.865, p = 0.001). No significant difference was found between Week 3 and Week 4 (Mean Difference = 1.055, p = 0.294). Figure 2 shows the comparisons of CMJ between groups across the different assessment moments.

For SJ, Mauchly’s test indicated that the assumption of sphericity was violated (W = 0.194, χ^2^ (5) = 58.56, p < 0.001); therefore, Greenhouse–Geisser corrections were applied (ε = 0.534). The repeated measures ANOVA revealed a significant main effect of time, F (1.60, 59.31) = 46.43, p < 0.001, partial η^2^ = 0.557, and a significant time × group interaction, F (1.60, 59.31) = 5.13, p = 0.014, partial η^2^ = 0.122. For the 2×/week training group, there was no significant difference in SJ height between Week 1 and Week 2 (Mean Difference = −0.011, p = 1.000), Week 1 and Week 3 (Mean Difference = −0.442, p = 0.993), or Week 2 and Week 3 (Mean Difference = −0.432, p = 0.251). However, performance declined significantly between Week 1 and Week 4 (Mean Difference = 2.953, p < 0.001), Week 2 and Week 4 (Mean Difference = 2.963, p < 0.001), and Week 3 and Week 4 (Mean Difference = 3.395, p < 0.001), with Week 4 showing lower SJ height. For the 3×/week training group, there was no significant difference between Week 1 and Week 2 (Mean Difference = 0.455, p = 0.146). SJ height decreased significantly from Week 1 to Week 3 (Mean Difference = 0.885, p = 0.037) and from Week 1 to Week 4 (Mean Difference = 2.310, p < 0.001). No significant change was observed between Week 2 and Week 3 (Mean Difference = 0.430, p = 0.226), but SJ height declined significantly from Week 2 to Week 4 (Mean Difference = 1.855, p = 0.001). No significant difference was found between Week 3 and Week 4 (Mean Difference = 1.425, p = 0.051). Figure 2 shows the comparisons of SJ between groups across the different assessment moments.

For participants training 2×/week, the average DOMS scores remained relatively consistent and low across the measurement weeks. In week 2, the average DOMS was 1.76 with a standard deviation of 0.54. This slightly decreased to an average of 1.59 (SD = 0.69) in week 3. By week 4, the average DOMS was 1.63, with a standard deviation of 0.67. In contrast, participants training 3×/week experienced generally higher average DOMS scores and greater variability. In week 2, the average DOMS was 2.19 (SD = 1.01). The peak average DOMS for this group was observed in week 3, reaching 2.44, with a standard deviation of 1.05, suggesting a wider range of DOMS experiences. By week 4, the average DOMS for the 3x week group decreased to 1.75, with a standard deviation of 0.88, bringing it closer to the levels observed in the 2x week group.

**FIGURE 3:**
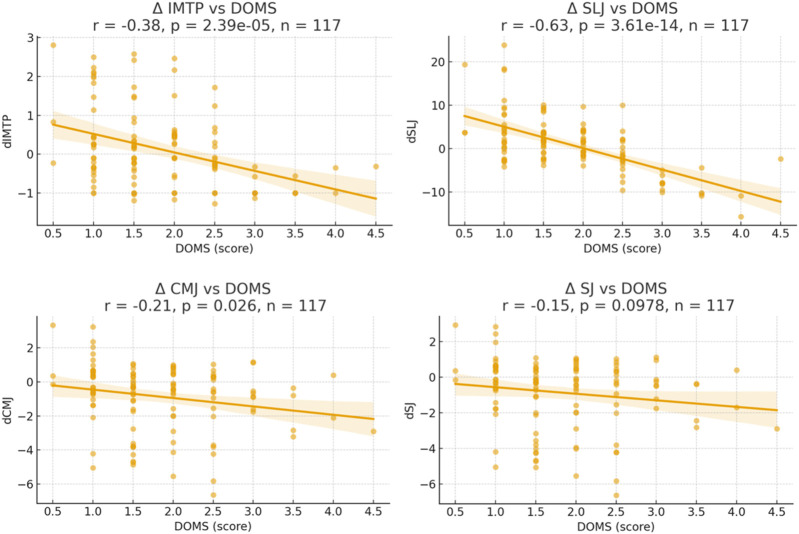
Scatter plots showing the correlations and 95% confidence intervals between the median delayed onset muscle soreness (DOMS) scores over the weeks and the median week-to-week changes in isometric mid-thigh pull (IMTP), countermovement jump (CMJ), squat jump (SJ), and standing long jump (SLJ) performance.

Significant negative associations were observed between DOMS and changes in IMTP (r = −0.38, 95% CI [–0.53, −0.21], p < 0.001) and SLJ (r = −0.63, 95% CI [–0.73, −0.50], p < 0.001), indicating that higher soreness scores were related to reduced maximal strength and horizontal jump distance. A weaker but significant negative correlation was also found for CMJ (r = −0.21, 95% CI [–0.37, −0.03], p = 0.026), whereas no significant relationship was detected for SJ (r = −0.15, 95% CI [–0.33, 0.03], p = 0.098). Scatterplots with regression lines illustrating these associations are presented in Figure 3.

## Discussion

Increasing training to 3×/week was associated with moderate-to-large decreases in IMTP (η^2^_p_ = 0.417) and SLJ (η^2^_p_ = 0.309) performance, alongside higher reported DOMS; these measures later returned toward baseline during the taper. In contrast, the twice-weekly training group showed steadier performance in both IMTP and SLJ, with small between-week fluctuations (IMTP mean change from Week 1 to Week 3: −0.31 N.kg^-1^, 95% CI [–0.9, 0.2]). By Week 4, when both groups were tapering at two sessions per week, performance in the 3×/week group had recovered, while the 2×/week group showed no further gains. Thus, although group differences converged in the taper, the interaction effects highlight that higher frequency was associated with transient but practically meaningful decrements in neuromuscular function during intensified phases.

There was a significant decline in SJ and CMJ height performance during the third evaluation (following the second week of intensification) in the 3×/week group, with performance significantly worse than in the 2×/week group (Week 3 CMJ mean difference: −1.25 cm, 95% CI [–2.1, −0.4]). A within-group impairment was observed as well, with performance in 3x week significantly lower than at baseline and the final evaluation after tapering. A previous study (Watkins et al., 2017) found reductions in vertical jump height following resistance training, which were accompanied by declines in neuromuscular function. The significant decline in SJ and CMJ performance during the third evaluation, following the second week of intensified training (3×/week), may be consistent with accumulated neuromuscular fatigue, which likely impaired both central motor drive and peripheral muscle function (Carroll et al., 2017), reducing the ability to generate explosive power (Mackey et al., 2018). Decreased neural efficiency, due to heightened central nervous system inhibition during intense training periods, could also limit motor unit recruitment and coordination (Carson, 2006). Importantly, impairments in neuromuscular capacity are known to emerge before measurable decrements in jump height, with technical alterations in movement patterns often evident at earlier stages (Ruggiero et al., 2022). Thus, when a clear decline in jump height is observed, this typically suggests that neuromuscular fatigability is already pronounced and that athletes may be experiencing transient performance decrements consistent with increased fatigue, as previously reported in elite populations (Gathercole et al., 2015; Ruggiero et al., 2022). Additionally, hormonal imbalances—particularly elevated cortisol levels from training stress—may have disrupted recovery and muscle protein synthesis (Angeli et al., 2004). In turn, the subsequent tapering phase and enhanced recovery likely allowed these systems to rebalance, explaining the post-taper performance improvement.

Participants training 3×/week experienced performance impairments during Weeks 2 and 3 (the intensified phase). By Week 4, when both groups reduced to two sessions per week during the taper, performance in the 3×/week group returned to baseline levels. This convergence should be interpreted as a recovery effect resulting from the reduced training load, rather than an adaptation to the higher-frequency phase. No further improvements were observed in either group during this period. Although these athletes experienced short-term decrements, the between-group analysis revealed no meaningful differences in IMTP values compared to those training twice per week. Taken together, these findings suggest that, over the short term, increasing plyometric frequency to three sessions per week does not provide additional performance benefits beyond those observed with two sessions. The literature remains mixed on this issue: while some studies (Palma-Muñoz et al., 2021) report progressive PT strategies yielding benefits after six weeks—particularly for jumping performance—other research has found that greater training frequency does not necessarily produce superior adaptations compared to lower frequencies (Ramírez-Campillo et al., 2015a; Ramirez-Campillo et al., 2018).

IMTP can be influenced by factors such as neuromuscular adaptation and recovery. Research indicates that increased training frequency can lead to heightened neuromuscular fatigue, particularly during intensified training phases (Roe et al., 2017; Lievens et al., 2021). This fatigue can impair force production due to temporary disruptions in neural drive and muscle contractility (Pethick and Tallent, 2022). Specifically, during the intensified weeks, increased training loads likely exacerbated neuromuscular fatigue and reduced recovery time, leading to temporary decrements in performance (Jones et al., 2016). The lack of significant improvements in the tapering phase for both the two- and three-sessions-per-week groups suggests insufficient recovery or adaptation from the intensified phases, which aligns with findings that excessive frequency without adequate recovery can impair long-term performance gains (Kellmann et al., 2018). However, it is also important to recognize that the present intervention lasted only 4 weeks, which may be too short a timeframe to observe the cumulative benefits or delayed adaptations associated with higher training frequencies. Longer interventions might reveal whether transient impairments observed during intensified training eventually translate into superior adaptations once adequate recovery is provided.

Regarding SLJ performance, our results indicate a significant main effect of time and a group-by-time interaction. Specifically, the group training twice per week showed consistent improvements in SLJ distance by Week 4. In contrast, the group training 3×/week experienced an initial decrement in SLJ performance, which subsequently recovered and increased by the end of the intervention. This suggests a potential acute overreaching effect with higher plyometric frequency (Carroll et al., 2017), impacting horizontal jump capabilities. Furthermore, the significant negative correlation observed between DOMS and SLJ performance highlights that increased muscle soreness was associated with reduced SLJ outcomes, underscoring the importance of recovery in maintaining horizontal power expression (Mackey et al., 2018).

Our findings indicate that higher DOMS scores were associated with week-to-week fluctuations in IMTP and SLJ performance. These exploratory correlations should not be interpreted as evidence of causality but rather as preliminary associations that warrant further investigation. Because multiple outcomes were examined, the results should be interpreted with caution and regarded as hypothesis-generating. The group training 3×/week consistently reported higher average DOMS scores, with a peak in Week 3, compared to the twice-weekly group. Across the sample, DOMS was negatively correlated with both IMTP changes and SLJ performance, suggesting that greater muscle soreness coincided with reduced maximal isometric strength and horizontal jumping ability. These findings are consistent with prior reports that eccentric loading—characteristic of plyometric exercise—can induce DOMS (Jamurtas et al., 2000), which in turn has been linked to temporary reductions in isometric strength and range of motion (Cleak and Eston, 1992).

This study has several limitations that should be acknowledged. First, the relatively short 4-week intervention may not fully capture longer-term adaptations or cumulative effects of different plyometric frequencies. Second, the non-randomized observational design limits causal inference, as group allocation followed the natural periodization decisions of athletes’ coaches rather than random assignment. Consequently, potential confounding variables such as concurrent strength or technical training, recovery modalities, sleep, and nutrition were not systematically monitored, and residual confounding cannot be ruled out. Third, although reductions in CMJ and SJ were observed, these findings should be interpreted cautiously, as the decrements were modest, not consistently significant across time points, and may reflect transient fluctuations rather than robust impairments. Fourth, DOMS was assessed using a modified 0–6 Likert-type scale which, while practical in the applied setting, is less conventional than the commonly used 0–10 scale and remains a subjective measure of muscle discomfort. Fifth, multiple outcomes (IMTP, CMJ, SJ, SLJ) were analyzed as co-primary endpoints to capture complementary aspects of neuromuscular performance. While Bonferroni adjustments were applied to *post hoc* comparisons within each outcome, the use of several parallel endpoints increases the risk of Type I error across outcomes and should be considered when interpreting the results. Sixth, the study sample consisted exclusively of regionally competing male jumpers aged 17–23, which restricts the generalizability of the findings to female athletes, other age groups, sports, or competitive levels. Finally, although the testing sequence and recovery intervals were standardized across all participants and sessions, subtle order, learning, or fatigue effects cannot be completely excluded.

Future research should employ randomized controlled designs, extend intervention durations, and include a wider range of athletic populations to strengthen external validity. In addition, the use of objective biomarkers and standardized recovery measures could provide a more comprehensive understanding of training frequency effects. Finally, more detailed analyses of the force–time characteristics obtained from jump force traces—including impulse development, rate of force production, and force profile changes—represent an important avenue for future work.

Despite the limitations, the present study highlights the value of monitoring both subjective and objective markers to guide PT frequency in jump athletes. In practical terms, week-to-week tracking of DOMS and performance provides coaches with some thresholds for decision-making. For example, if DOMS ratings reach ≥2 (mild soreness noticeable but not disabling) and SLJ performance decreases by ≥ 5% relative to baseline, coaches may consider postponing or reducing the volume of the third weekly PT session to avoid excessive fatigue accumulation. Conversely, when DOMS remains ≤1, SLJ and CMJ performance are stable or improving, maintaining or progressing to three sessions per week may be appropriate. By combining monitoring tools (DOMS scale, and weekly jump testing), coaches can make individualized, data-informed decisions to balance training frequency, adaptation, and recovery.

## Conclusion

A twice-weekly PT frequency appears to be more effective in promoting consistent yet modest improvements in IMTP and SLJ over a 4-week period, while minimizing acute fatigue. Conversely, increasing training to 3×/week was associated with greater short-term fatigue, higher DOMS, and modest decrements in jumping performance, which appeared transient—particularly in IMTP and SLJ—before subsequent recovery. The observed negative correlations between DOMS and these performance measures suggest the role of recovery in optimizing training outcomes. These findings highlight the importance of carefully considering training frequency and monitoring athlete readiness to maximize the benefits of PT and prevent overreaching in jumping athletes.

## Data Availability

The raw data supporting the conclusions of this article will be made available by the authors, without undue reservation.
